# Acceptability and feasibility of acceptance and commitment therapy for improving outcomes in hematopoietic stem cell transplant

**DOI:** 10.1371/journal.pone.0319339

**Published:** 2025-03-07

**Authors:** Rhonda M. Merwin, Patrick J. Smith, J.A. Riley, Jordan Infield, Christine O’Connell, Dorothy Mayo, Ashley A. Moskovich, Lauren Hill, Hilary Winthrop, Amy Bush, Ernaya Johnson, Francesca Scheiber, Anthony D. Sung

**Affiliations:** 1 Department of Psychiatry and Behavioral Sciences, Duke University School of Medicine, Durham, North Carolina, United States of America; 2 Department of Psychiatry, University of North Carolina at Chapel Hill, Chapel Hill, North Carolina, United States of America; 3 Duke Center for Aging, Duke University School of Medicine, Durham, North Carolina, United States of America; 4 Department of Medicine, Duke University School of Medicine, Durham, North Carolina, United States of America; 5 Department of Medicine, Division of Hematologic Malignancies and Cellular Therapy, Duke University School of Medicine, Durham, North Carolina, United States of America; 6 Duke Office of Clinical Research, Duke University School of Medicine, Durham, North Carolina, United States of America; Mayo Clinic College of Medicine and Science, UNITED STATES OF AMERICA

## Abstract

**Introduction:** Allogeneic hematopoietic stem cell transplant (HCT) has the potential to cure patients with hematologic malignancies, but treatment-related morbidity and mortality is high. Transplant outcomes are optimized by patients maintaining physical activity. The aim of the current study was to examine whether a brief Acceptance and Commitment Therapy (ACT) intervention is acceptable to HCT patients and caregivers and helps patients engage in healthy behavior despite physical and emotional discomfort.

**Methods:** Patients ≥ 18 years of age who were undergoing allogenic HCT for any cancer or non-cancer illness and their caregivers were invited to complete six ACT sessions between transplant day − 30 and day + 90. Multiple small cohorts of n = 3 dyads were enrolled, and the protocol content was iterated after each cohort to reflect the experiences and breadth of concerns of individuals undergoing HCT. Acceptability was indexed by session completion rates and acceptability surveys. Pre-post 6-minute walk distance was collected as an index of physical function as part of standard care.

**Results:** Sixteen HCT dyads enrolled in the study; 12 continued to treatment. Most participants completed all assigned sessions. Participants perceived ACT to be helpful and 70% (5 of 7) of the patients with pre-post 6-minute walk test data showed improvement.

**Conclusion:** ACT is an acceptable and potentially useful intervention for individuals undergoing HCT. Additional controlled studies are warranted.

## Introduction

Allogeneic hematopoietic stem cell transplant (HCT) has the potential to cure patients with hematologic malignancies and other diseases. However, the treatment course is arduous and treatment-related mortality ranges from 10% to 30% [1–4]. Anxiety, depression and post-traumatic stress are common among individuals undergoing HCT and impact treatment adherence and treatment tolerance [5–7]. Caregivers of individuals with HCT also experience significant caregiver strain and diminished psychological health and well-being [8], which could influence their capacity to manage caregiver demands.

Prior studies of psychological interventions for HCT are limited [9,10]. In a 2016 meta-analysis, cognitive-behavioral therapy (CBT) interventions generally had the greatest impact; however, effect sizes were small, and studies with larger effects lacked methodological rigor [11]. Additionally, past studies have primarily focused on decreasing distress and improving quality of life for HCT patients or caregivers during the post-transplant period [12–14], with a few exceptions (e.g., [15]). When studies have engaged patients and caregivers pre-transplant (e.g., [16]), they have focused on improving caregiver distress and quality of life rather than building patient resiliency to treatment or increasing capacity to follow treatment recommendations that would optimize transplant outcomes (e.g., daily physical activity [17]).

Studies have also been plagued by low participation, due to the significant and unique burdens of transplant, which has led investigators to greater integration with routine care and remote delivery options [18]. A recent pilot randomized controlled trial [19] demonstrated that HCT patients can be engaged remotely to increase physical activity, but more work is needed to determine the best strategy for intervention.

Acceptance and Commitment Therapy (ACT) is a contemporary evidence-based CBT that improves human functioning and adaptability by increasing psychological flexibility (or the ability to behave flexibly and effectively in the presence of any and all thoughts and feelings, guided by personal values) [20]. Unlike traditional CBT, which emphasizes cognitive challenging and change, ACT teaches individuals to have an open and receptive posture with respect to moment-to-moment experiences and orient to personal values (i.e., what is most important to the individual and how they want to live the moments of their lives) to motivate and guide behavior. ACT may be particularly well-matched to HCT: a situation in which patients are likely to experience distressing thoughts/feelings that are *not* illogical or irrational (e.g., awareness of possible threat to life) and other unavoidable treatment-related discomforts (e.g., fatigue, pain, nausea). In the context of HCT, ACT may be leveraged to help patients be flexible and continue to approach, rather than avoid, eating and physical activity, despite the pain, discomfort or emotional distress that they experience during the pre-transplant preparative regimen/conditioning and post-transplant recovery period.

ACT has been used with patients with life-threatening diseases, including cancer, in previous studies and has been found to be beneficial [21–24]. In a recent meta-analysis, ACT was also associated with improvements in chronic fatigue and pain among those struggling with advanced cancer [25]. However, similar to traditional CBT studies, ACT studies in cancer have focused on improving quality of life rather than using ACT to increase adherence to cancer treatment recommendations to optimize outcomes [21–23].

Caregivers are also critical members of the treatment team and have the challenging task of helping the patient follow recommendations for maintaining nutrition and exercise during chemotherapy/radiation, when patients may experience severe discomfort or transient impairments in cognitive and emotional faculties, and during the posttransplant period. Including caregivers in psychological intervention is known to be helpful when individuals are physically and cognitively impaired, or in acute phases of illnesses which require feeding and activity management, such as in the case of anorexia nervosa [26–28].

While caregivers are central in optimizing treatment outcomes, most studies have focused on the impact of caregiving on mental health and well-being [15,29]. While this is important, it is different than psychological intervention focused on helping caretakers flexibly engage in caretaking when it is emotionally challenging (e.g., being present and supporting their loved one even in the presence of fear and sadness). Increasing psychological flexibility might help caregivers approach uncomfortable situations and be more effective in facilitating eating or physical activity in the moment. Greater caregiver effectiveness and less avoidant coping could have the dual benefit of: 1) improving transplant outcomes, and 2) decreasing the burden of caregiving, which has been associated with developing sleep issues, higher blood pressure, reduced immune system functioning, increased psychological distress [26] and poorer quality of life [30]. ACT may also potentiate the positive effects of caregiving, such as a greater appreciation of life and sense of purpose. In a few studies of ACT for caregivers of cancer patients, caregivers reported an increased ability to accept and cope with negative emotions and experiences, as well as a better understanding of their own values and ability to practice self-kindness and feeling closer with the patient [31].

The overarching aim of this Stage 1 study was to develop an ACT intervention for adults undergoing HCT and their caregivers with the ultimate goal of optimizing transplant outcomes. The protocol was based on the psychological inflexibility model and provided ACT skills to help individuals undergoing HCT maintain nutrition and physical activity pre- and post-transplant, despite pain, fatigue and emotional distress.

To our knowledge, this is the first study on using ACT to help individuals undergoing HCT maintain physical function despite treatment-related discomforts, which may have implications for posttransplant survival. It is also novel in its inclusion of the caregiver for the purpose of increasing caregivers’ psychological capacity to facilitate eating and physical activity.

The primary hypothesis was that the protocol would be acceptable to the majority of HCT patients and caregivers, as indicated by session attendance and acceptability surveys. Secondary outcomes explored signals of efficacy with measures of psychological flexibility and pre-post HCT physical function.

## Methods

This study was approved by the Ethics Committee of Duke University Medical Center and registered on clinicaltrials.gov (NCT 04423939). All participants provided written informed consent prior to enrollment in the study. This research was conducted ethically in accordance with the World Medical Association Declaration of Helsinki. Participants were not compensated for their participation.

### Enrollment

Participants were recruited between August 2020 and September 2022 from a single adult bone marrow transplant clinic at a large tertiary academic medical center in the Southeast United States. Patients ≥ 18 years of age who were undergoing allogenic HCT for any cancer or non-cancer illness were approached by a member of the study team based in clinic and invited to participate. To be eligible, patients had to have a caregiver who was willing to attend sessions and who also consented to participation, and both patient and caregiver had to be able to write and speak in English.

### Intervention

Individuals undergoing HCT and their caregivers were asked to complete six 45–60 minute conjoint ACT sessions between transplant Day − 30 (30 days before transplant) and Day + 90 (90 days after transplant).

ACT sessions aimed to increase psychological flexibility, such that patients and caregivers were able to maintain recommendation for diet and exercise in the face of uncertain and difficult treatment course, side effects and complications. Sessions included skill building using 6 inter-related ACT processes to increase psychological flexibility: acceptance, defusion, self-as-context, present moment awareness, values and committed action [32]. Acceptance was used to increase openness to internal experiences, including unwanted thoughts, feelings and body sensations, such that individuals could have experiences without needing to avoid or escape from them. Defusion was used to decrease overattachment to the content of mental activity (or create distance between the individual and their internal experiences), such that internal experiences, like the thought “I can’t stand this,” did not have undue influence over behavior. Self-as-context was used to increase the extent to which individuals experienced themselves as “more than” or “bigger than” the thoughts/feelings that they could observe and to help individuals flexibly take perspective. Present moment awareness was used to promote flexible attention and increased one’s capacity to be in the here-and-now, rather than caught up in thoughts about the feared future or regretted past. Values and committed action interventions were used help individuals clarify what was most important to them or how they wanted to live the moments of their lives (including the moments that are the most difficult). Committed action helped individuals align behavior with their chosen values.

The initial protocol (referred to as “ACTivate”) was drafted based on the psychological inflexibility model and expertise of the study team, which included a HCT physician, transplant health psychologist and clinical psychologist with expertise in ACT. The protocol was then implemented with a small cohort of participants (n = 3 dyads) and the content was expanded to address the breath of concerns of individuals undergoing HCT. The core protocol and skills were not changed, but the skill presentation and discussions were refined to better match the experiences of patients and caregivers. This process was repeated with an additional cohort (n = 3), and by the third administration of the protocol, no new issues or concerns emerged. The protocol was then trained to a new therapist and implemented with a final cohort (n = 3 dyads), for a total N = 12.

### Assessments

Acceptability of the intervention was assessed via session completion rates and acceptability surveys completed by patients and caregivers after session. Exploratory analyses examined change in psychological flexibility (the ability to pursue valued goals in the presence of distressing thoughts/feelings) over time. Change in weight and physical function (as indexed by a 6-Minute Walk Test), were also explored as a signal of intervention efficacy. Survival/mortality rates were recorded for reporting purposes.

### Acceptability surveys

Participants received a REDCap link to complete an acceptability survey online after each session, followed by 3 daily reminders if incomplete. Surveys consisted of the following items rated on a 5-point Likert scale (1 = Strongly Disagree to 5 = Strongly Agree): I liked the session; The session was helpful for managing difficult thoughts or feelings; The session was helpful for making behavioral changes; and I would recommend this session to a friend going through a similar experience. Participants were also provided with a free text item to provide general feedback, including anything they liked, didn’t like, or would change about the session. Specifically, they were prompted “Your feedback is extremely valuable and will help us determine how to best help other people going through a similar experience. Please provide your candid feedback about what you liked, did not like or would change about the session. Please be as specific as possible”.

After Session 6, participants received a REDCap link to a final acceptability questionnaire, and responded to the following items using the same Likert scale (1 = Strongly Disagree to 5 = Strongly Agree): I liked the program; The program was helpful for managing difficult thoughts or feelings; The program was helpful for making behavioral changes; I would recommend this program to a friend going through a similar experience, as well as a free text response for any additional feedback.

### Psychological flexibility

Psychological flexibility was assessed with the Valuing Questionnaire (VQ) [33]; which has been found to be more psychometrically sound than other commonly used measures, such as the Acceptance and Action Questionnaire (AAQ) [34]. The VQ is a 10-item scale consisting of two subscales: Values Obstruction and Values Progress. Only the Obstruction subscale was used in the current study, which consists of 5 items that assess the extent to which thoughts/feelings interfered with behaving in accordance with personal values over the past week [33]. Items in the current study used a 6-point Likert scale ranging from (1 = Not at All True to 6 = Completely True), which slightly deviates from the original 7-point scale (0 = Not at All True to 6 = Completely True). Sample VQ Obstruction items include: “I spent a lot of time thinking about the past or future, rather than being engaged in activities that mattered to me.”; “Difficult thoughts, feelings or memories got in the way of what I really wanted to do.”; “When things didn’t go according to plan, I gave up easily.” Lower scores reflect less values obstruction (or greater psychological flexibility).

Participants received a REDCap link to complete the VQ at baseline (study enrollment, up to − 60 Days prior to transplant) and every 2 weeks through Day 30 post-transplant. They completed the VQ again at Day + 60 and Day + 90.

### Height and weight measurements

Patients’ height and weight were measured at the New Patient Evaluation for transplantation (−180 to − 30 Days prior to transplant) and just prior to start of chemotherapy/radiation (−7 to − 14 Days prior to transplant). Weight measurements were also taken at Day + 30 and Day + 90 post-transplant as part of standard clinic protocol*.* Weight loss is common among patients who receive HCT with studies showing a median decrease of 6.6% in body mass index (BMI) from admission to discharge [35].

### 6-Minute walk test (6MWT)

Patients completed a 6-Minute Walk Test (6MWT) at a New Patient Evaluation for transplantation (−180 to − 30 Days prior to transplant) and just prior to the start of chemotherapy/radiation (−7 to − 14 Days prior to transplant), Day 30 and Day 90 post-transplant, as part of the standard clinic protocol. The 6MWT provides an index of physical function pre- and post-transplant [36,37]. In the 6MWT, individuals are asked to walk consistently for 6 minutes while distance is measured. The 6MWT provides important data about activities of daily living and the process of recovery and maintenance of physical function after a procedure like HCT. 6MWT distances typically decline over the transplant period, although ideally functioning would be maintained. For example, a recent study reported average 6MWT distances for HCT patients were 431 meters at baseline, 400 meters at 30 days post-transplant, and 408 meters at 90 days post-transplant [38].

### Data analysis

Acceptability data, including sessions attended and individual responses to participant surveys, were summarized with descriptive statistics (mean, *SD*). Qualitative responses from caregivers and patients were compiled for reporting. Descriptive statistics (mean, *SD*) and difference scores were also calculated for exploratory process and outcome measures. All data were individual. SAS 9.4 was used to produce descriptive statistics.

### Data sharing

Primary data generated or analyzed during this study are included in their entirety in this published article itself. Remaining data are protected health information (PHI).

## Results

### Participants

**Fig 1:**
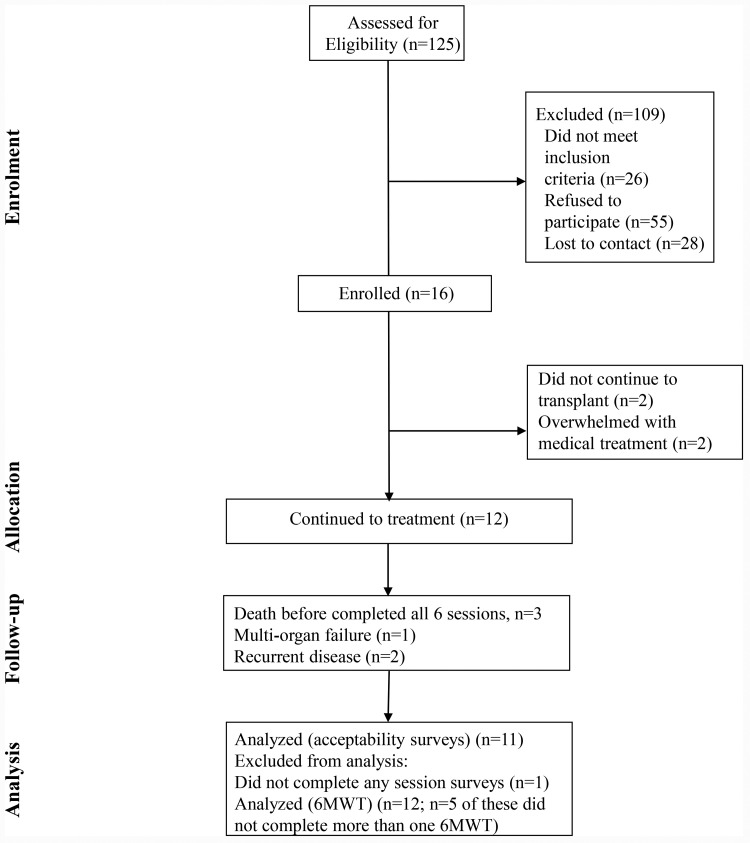
CONSORT Statement Flow Diagram.

Thirty-two individuals, or 16 dyads (16 HCT patients and their 16 caregivers), enrolled in the study between October 2020 and June 2022 (See Fig 1 and Table 1). Of the 16 dyads, 12 dyads continued to treatment. Four dyads withdrew after enrolling due to changes in the treatment plan (no longer undergoing transplant) (n = 2) or feeling overwhelmed with medical treatment (n = 2). All the patients who withdrew were White males. No additional data were collected on participants who withdrew before treatment, and they were not included in any analyses.

**Table 1:** Baseline Patient and Caregiver Characteristics, Transplant Diagnosis and Conditioning Regimen.

	Enrolled Patients (*N* = 16)	Patients who Received ACTivate and HCT *(N* = 12)	Caregivers of Patients who Received Activate and HCT (*N* = 12)
Baseline Demographics and Clinical Measure**s**			
Age at transplant (yrs), mean (*SD*)	59.4 (9.9)	52.8 (15.9)	56.2 (16.5)
Male sex, *n* (%)	9 (56%)	5 (42%)	6 (50%)
White race, *n* (%)	12 (75%)	9 (75%)	8 (67%)
Hispanic/Latinx	1 (6.3%)	1 (8.3%)	0 (0%)
Body Mass Index (BMI) at baseline prior to transplant, mean (SD)		26.7 (3.5)	
Transplant Diagnosis, *n* (%)			
Acute Myelogenous Leukemia (AML)	8 (50%)	4 (33.3%)	
Acute Lymphoblastic Leukemia (ALL)	1 (6.3%)	1 (8.3%)	
Chronic Myelogenous Leukemia (CML)	1 (6.3%)	1 (8.3%)	
Myelodysplastic Syndrome (MDS)	4 (25%)	2 (16.7%)	
Lymphoma	1 (6.3%)	1 (8.3%)	
Other	2 (12.5%)	3 (25%)	
Conditioning Regimen			
Myeloablative, *n* (%)	7 (43.8%)	7 (58.3%)	
Reduced intensity, *n* (%)	0 (0%)	0 (0%)	
Non myeloablative, *n* (%)	5 (31.3%)	5 (41.7%)	

The mean age of the individuals who continued to treatment was 52.8 years. Seventy-five percent of patients were White and 8% identified as Hispanic or Latinx. Forty-two percent were male. The most common transplant diagnosis was acute myelogenous leukemia (AML) (33%), followed by myelodysplastic syndrome (MDS) (17%). There was an equal representation of diagnoses of acute lymphoblastic leukemia (ALL), chronic myelogenous leukemia (CML), and lymphoma (each comprising 8% of the sample). Over half of the HCT patients (58%) received myeloablative conditioning. The caregivers were spouses (*n* = 7), immediate family members (*n* = 2 mother, *n* = 1 father, and *n* = 1 sister), and a friend (*n* = 1).

### Protocol content

ACT is a transdiagnostic intervention and the core processes of psychological flexibility could be applied to this population as with others. However, the current study aimed to apply ACT skills to the unique experiences and needs of HCT patients and caregivers, and address barriers to engaging in health behaviors (e.g., physical activity) that optimize transplant outcomes. The full protocol is outlined in Table 2. Initial skill discussions focused on anticipated issues, such as physical discomfort associated with chemotherapy, and inactivity and withdrawal related to the emotional pain of having a life-threatening illness. However, following each of the first two administrations of the protocol, the skill discussions were also expanded to address the breadth of concerns of individuals undergoing HCT identified during open-ended conversations in sessions.

**Table 2:** Overview of the ACTivate Protocol.

	Activities	Description
Session 1 Target date for completion: − 30 days (30 days before transplant)	Introductions; Goals and structure of the program	Introduction to the counselor and the goal of the program to increase psychological flexibility (the ability to behave flexibly in the presence of difficult thoughts and feelings) in the service of optimizing transplant outcomes.
	Your north star(s) (identifying personal values)	Clarification of patient and caregiver’s personal values that can guide actions in difficult times.
	The challenge and gift of the ever-evolving treatment plan	Discussion of the dynamic and uncertain nature of the situation using a Journey Metaphor. This metaphor helps people open up to the uncertainty of the transplant process and the unexpected turns that will arise, and foster resiliency to overcome any barriers.
	Your job/ The caregiver’s job	Identifying the different roles of the patient and caregiver and the importance of nutrition and physical activity (under various uncomfortable conditions and in the presence of distressing and unwanted thoughts and feelings) for transplant outcomes.
	Home Practice: Write about personal values	Invitation to continue to explore personal values through writing.
Session 2 Target date for completion: − 30 to – 15 days (30 to 15 days before transplant)	Acknowledging thoughts and feelings	Identification of patient and caregiver’s difficult thoughts/feelings about the illness or treatment.
	Passengers on the bus	Passengers on the Bus Metaphor is used to open up to thoughts/feelings as “passengers” (experiences that can “come along”), rather than the “driver” (that determines actions). The metaphor also highlights the individual as the observer of experience or a person that as “more than” or “bigger than” any thought/feeling.
	Set a SMART goal	Setting a goal that is specific, measurable, action-oriented, realistic and time-based for improving or continuing eating and physical activity during conditioning, transplant and post-transplant (revisited as needed).
	Home Practice: Passengers on the bus worksheet. Set and practice your SMART goal.	Dyad is invited to complete the Passengers on the Bus worksheet to solidify concepts and practice their SMART goal, connected to personal values.
Session 3 Target date for completion: − 15 to 0 days (15 to 0 days before transplant)	The things you can’t control	Discussion of when intentional control is helpful (e.g., controlling behavior or actions) and when it is not (e.g., controlling or suppressing thoughts and feelings) and acknowledging the limits of control in this unique situation.
	Short-term relief and long-term costs	Discussion of how some behaviors or actions might provide relief from discomfort in the short-term, but may be unhelpful in the long-term or have costs for personal values. Dyad explores their own patterns of avoidance.
	Open up to “guests” in your home	Guests in Your Home Metaphor is used to help the patient and caregiver allow uncomfortable emotions to behave flexibly in line with values. The metaphor highlights the dynamic nature of emotions and how they can be welcomed as visitors. This metaphor also helps the patient and caregiver experience themselves as more than feelings they may have.
	Unhook from unhelpful thoughts	The metaphor of being hooked by thoughts is introduced. The patient and caregiver identify their “hooks” (e.g., thoughts that unduly influence their behavior) and are encouraged to “unhook” (observe these thoughts as mental activity from a more distant vantage point) when it serves their values.
	Attention is a spotlight	Attention is described as a spotlight (that highlights different aspects of experience). The patient and caregiver consider where they direct their attention and issues of vigilance, avoidance, fixed or narrow attention. The aim is broad and flexible attention (the ability to take in a situation more fully and use information to guide action adaptively).
	Home practice: Practice unhooking, welcoming unwanted feelings. Smart goals.	The dyad is invited to practice strategies from session at home and to continue with SMART goals.
Session 4 Target date for completion: 0 to + 15 days (0–15 Days after transplant)	Being where you are and present moment awareness	The Time Traveling Metaphor is used to discriminate when the patient’s or caregiver’s mind is in the present, future or past,and present moment awareness skills are practiced (e.g., mindful breathing) to help the patient and caregiver be more present when it serves their valued goals.
	Doing the next right thing	The idea of choice points is introduced and the patient and caregiver explore the idea of making a choice that serves their valued goals in the moment with a process rather than outcome orientation.
	When your mind is full, get mindful	The patient and caregiver practice discriminating the mind being “full” (of ideas, to do lists etc.) versus mindful awareness of one’s surroundings.
	Home practice: Present moment awareness exercises. SMART goals.	The dyad is invited to practice strategies from session at home and to continue with SMART goals.
Session 5 Target date for completion: + 15 to + 45 days (15–45 days after transplant)	Living your values	Discussion returns to personally meaningful values and how values may be lived in daily life (and compassionately under any and all circumstances).
	Predicting the hooks and staying on course (or returning)	The patient and caregiver identifies difficult thoughts/feelings that are here now, or that they are likely to experience in the future and how they might use skills to stay on course with eating, activity and other valued goals.
	Home practice: Valued intention. Smart goals	The dyad is invited to practice strategies from session at home and to continue with SMART goals.
Session 6 Target date for completion: + 45 to + 90 (45–90 days after transplant)	Living with intention	Living each day with intention (rather than on “autopilot”) is discussed. The patient and caregiver consider how they might live fully each day, given any physical limitations, recovery time or complications.
	Making meaning	The patient and caregiver is invited to consider their meaning in this challenging experience, the ways in which they have changed and stayed the same and using values as a guide.
	Home practice: Value-guided behavioral activation	The dyad is encouraged to continue to build broader patterns of activity linked to personal values.

These included:

- Blind optimism: Some dyads described positive thinking that was inflexible (e.g., “Do not think about anything bad.”) and interfered with being adequately prepared for the challenges of HCT (psychologically or practically). They needed help being open to a broader range of experiences (and possibilities) to be more effective.
- Limits of control: Some dyads reported struggling with uncontrollable external circumstances (e.g., the experience of being connected to medical devices). Accepting the limits of control and feeling helpless was a central theme for these dyads.
- Recovery timeline: Some patients were distressed by the timeline of recovery, which felt too long. Some patients had difficulty engaging in the activities that they were able to now, while also pacing their return to other activities (i.e., they were either doing too much or too little). These individuals needed help balancing acceptance of their experience (in this moment) with change (taking action when action is possible).
- Relational issues: Some patients reported concern for other people (e.g., not wanting children to see them sick) or not wanting to be a burden to others, which interfered with engaging in their life more fully (e.g., video chats) or asking for needed support. These individuals needed help accepting these feelings to allow people to be there for them.
- Stage of life issues: Some patients needed help accepting the impact of illness and transplant on typical trajectories of life (e.g., the possibility of having children, returning to previous work vs retirement). These individuals needed help accepting loss, and letting go of ideas of what life was “supposed to be” to be more fully present and engaged with “what is.”
• Coping with the possibility of end of life. Some patients reported guilt and regret related to the possibility of end of life. These individuals needed help accepting themselves as fallible humans and living life now.

### Protocol delivery

The intervention was initially designed to be conducted in person and delivered at the time of the HCT treatment milestones. However, this study took place during COVID-19 and this population needed greater flexibility due to unpredictability of HCT and managing complications and emergencies. Thus, all sessions were conducted virtually by Zoom as close in time as possible to a target session date (See Table 1), based on patient’s medical status, availability, and ability to participate. Sessions were usually conjoint, although sometimes patients and caregivers were seen separately due to scheduling or the need to address an individual patient or caregiver issue.

### Acceptability

All participants completed at least 4 sessions and 75% completed all 6 sessions (M = 5.17 sessions, *SD* = 1.04). Noncompletion was due to continued illness (n = 1) or death (n = 2). Generally, patients were motivated to reschedule if something interfered with their attendance or to overcome challenges to attend.

While session attendance was high, survey completion rates were not optimized. Participants completed some surveys some of the time. This was may have been due to several factors reported by participants, including treatment-induced fatigue (e.g., patients reported that they fell asleep after session), transplant related treatment visits immediately following session and/or medical complications that resulted in unexpected treatment or hospital admission, among other reasons not reported.

Despite these challenges, a total of sixty-six acceptability surveys were completed by 11 different patients and 11 different caregivers. Ninety-two percent of the surveys indicated satisfaction with the session (patients and caregivers “Strongly Agreed” or “Agreed” that they liked the session), and 95% indicated that they would recommend it to a friend going through a similar experience. Regarding perceived helpfulness, on 88%–89% of the surveys (respectively), patients and caregivers “Strongly Agreed” or “Agreed” that the sessions helped them manage difficult thoughts or feelings and that the session was helpful for making behavioral changes. On the final acceptability survey, completed by 7 of the 11 living dyads, all “Strongly Agreed” or “Agreed” that they liked the program, that it was helpful for managing difficult thoughts or feelings and for making behavior changes, and that they would recommend the program to a friend going through a similar experience. Qualitative feedback provided by patients and caregivers is provided in Table 3. No adverse events related to the ACT intervention were reported by participants or caregivers.

**Table 3:** Qualitative Feedback from Patients and Caregivers on Session and Program.

Patient Feedback	Caregiver Feedback
“It seems that this will be helpful in our journey through this.”	“The [Counselor] made us feel comfortable and validated the emotions we are dealing with. She listened and incorporated things we are already doing while suggesting additional strategies to accomplish the same goals of the program.”
“I liked the use of metaphor to explain concepts, and tools to use when needing to center myself. The ease of talking to her and her ability to listen and understand.”	“For a first session it seemed good. Although I didn’t know what to expect.”
“I don’t think I would change anything. I expect future meetings to be even more helpful.”	“I particularly liked the analogy of feelings being like guests in your house. I also thought [Counselor] did an excellent job of listening and giving [spouse] suggestions of images of forgiving someone and then substituting a younger version of himself as a means of showing compassion and forgiveness of himself.”
“Hey the [session] help me through my emotions [.] I am trying hard to get up and walk every day, clean up [.] Just being myself I can do this [with] God help.”	“I appreciate that while these sessions have a goal they are flexible and responsive to my needs and situation. [Counselor] is excellent!.”
“I think since today was just the first session, there was more getting to know each other that was being done so I can’t 100% judge this session completely.”	“[Counselor] is great. It seems that her insight for how to approach each new aspect of the process was extremely intuitive, given necessary ‘take aways’ that add values to whatever gifts I was bringing to the caretaker experience.”
“I liked the breathing exercises and focusing on the 5 senses. It did lower my anxiety level. Now I need to remember to do it myself.”	“So thankful we participated! [counselor] is fabulous!”
“Great conversation with [Counselor], [spouse] and I. We appreciate her professional input.”	
“Very satisfied. Session was very helpful.”	
“I believe the study helped a lot. Paper copies would be great for future groups who are more hands-on than visual.”	
*Additional comments from patients and caregivers provided in other formats (e.g.,* *email communication)*	
“This study was extremely supportive and helpful as we’ve navigated this process.”	“I’m using communication for sharing information and eliciting discussion rather than for outcome of right or wrong. This has helped us so much to bump heads much less.”
	“I [the caregiver] used the passengers on the bus to help her [the patient] with the strong negative feelings and thoughts she was having at the setback.”
	“I also would like to say that it’s been great talking with [Counselor] while we are going through the transplant process. The people on the bus project has stuck with us throughout our time here and it’s really had a positive impact in our quest.”
	“There are many places where the burden of caretaking can foster communication that has negative consequences. The idea of giving the caretaker a toolchest of helpful hints is an invaluable asset, encouraging the caretaker to remain emotionally “present” and not “check out.”

### Psychological flexibility: Valuing questionnaire – obstruction subscale

Values Obstruction scores for patients and caregivers are presented in Table 4a-4b, respectively. Lower VQ scores reflect less values obstruction, or higher psychological flexibility, i.e., thoughts/feelings interfering less with engagement in valued activities. Seventy percent of patients indicated a decrease in values obstruction from baseline to last observation and overall mean change was negative. Caregiver scores were more mixed; 2 caregivers indicated a decrease in Values Obstruction from baseline to last observation, 3 reported an increase and 3 were unchanged (change of < 1 point).

**Table 4a:** Patient VQ-Obstruction Subscale Scores.

Patient #	Baseline	During Activate		Post Activate	Difference (Baseline – Last Observation)
		Before transplant	After transplant		
1	16.00	16.00	9.00	–	−7.00
2	7.00	–	–	–	–
3	15.00	7.00	10.00	6.50	−8.50
4	11.00	7.00	–	–	−4.00
5	7.00	0.00	3.50	2.00	−5.00
6	6.00	–	3.67	2.00	−4.00
7	8.00	0.25	1.67	0.50	−7.50
8	4.00	1.67	3.50	6.00	2.00
9	–	–	–	–	–
10	0.00	5.00	–	–	5.00
11	15.00	14.75	10.00	12.00	−3.00
12	11.00	–	15.00	–	4.00
Mean (*SD*)	9.09 (5.03)	6.46 (6.16)	7.04 (4.63)	4.83 (4.25)	−1.40 (5.47)

**Table 4b:** Caregiver VQ-Obstruction Subscale Scores.

Caregiver #	Baseline	During ACTivate		Post ACTivate	Difference (Baseline – Last Observation)
		Before transplant	After transplant		
1	–	–	7.00	–	–
2	–	–	–	–	–
3	–	1.00	0.50	0.00	–
4	4.00	2.00	7.33	5.00	1.00
5	5.00	–	5.00	5.00	0.00
6	4.00	6.00	4.00	7.00	3.00
7	9.00	18.25	14.67	14.50	5.50
8	5.00	5.00	5.50	–	0.50

VQ=Valuing Questionnaire; Baseline =  VQ score at study enrollment (up to −60 Days before transplant); Before Transplant =  mean of VQ surveys completed every 2 weeks from

Baseline to Day 0, After Transplant =  mean of VQ surveys completed on Day 14 and Day 30, Post

ACTivate =  mean of VQ surveys completed at Day 60 and Day 90, - =  missing.

### Clinical outcomes (patients only)

#### Weight.

Weight data are summarized in Table 5. At 30 Days, *n* = 7 gained weight relative to pre-transplant; by 90 days, the majority had lost weight. Weight loss ranged from 1.1 to 14.6 kg. Most patients lost between 2 and 5 kg (or 5–10lbs). A few (*n* = 3) lost 9–15 kg (20–30lbs). Patients’ BMI ranged from 21.98 to 32.89 at baseline. Patients who lost the most weight still had BMIs between 25.06 and 26.00. Two of the three patients with the most weight loss died within 1-year post-transplant (Patients 4 and 6).

**Table 5:** Weight (kg) at Baseline, 30 and 90 Days Post-Transplant.

	Baseline	D30	Difference (Baseline – D30)	D90	Difference (Baseline – D90)
1	89.1	90	0.9	–	–
2	83	71.1	−11.9	73	−10
3	75	75.9	0.9	73	−2
4	87	94.5	7.5	72.4	−14.6
5	56	57.8	1.8	53.4	−2.6
6	89	83.3	−5.7	80	−9
7	91	86.2	−4.8	88.5	−2.5
8	101.5	109.4	7.9	96.8	−4.7
9	66.3	64.7	−1.6	62.4	−3.9
10	66.4	62.6	−3.8	67	0.6
11	89	94.3	5.3	96.3	7.3
12	59.9	59.9	0	61	−1.1
Mean (*SD*)	79.43 (14.33)	79.14 (16.37)	−0.29 (5.76)	74.89 (14.27)	−3.86 (5.82)

kg=kilograms, SD=standard deviation, D30 = 30 days after transplant, D90 = 90 days after

transplant, - =  missing.

Baseline weight =  Weight taken during standard clinical procedures for transplantation, most often at the New Patient Evaluation (−180 to −30 Days prior to transplant). In cases where data from the New Patient Evaluation were missing, baseline is the patient’s weight prior to the start of chemotherapy/radiation (−7 to −14 Days before transplant).

### 6MWT

6MWT data are summarized in Table 6. The majority of participants walked a longer distance at 30 or 90 days post-transplant compared to baseline, suggesting that they maintained or improved physical function. Two patients walked a shorter distance at 30 and 90 (Patient 

7) or 90 (Patient 2) days. One of these individuals (Patient 7) died within one-year of transplant.

**Table 6:** Minute Walk Test (meters) at Baseline, 30 and 90 Days Post-Transplant.

	Baseline	D30	Difference (Baseline – D30)	D90	Difference (Baseline – D90)
1	442.67	–	–	–	–
2	523.89	532.99	9.1	498.64	−25.25
3	458.28	514.42	56.14	–	–
4	467.11	–	–	–	–
5	437.44	520.95	83.51	529.96	92.52
6	390	418.98	28.98	470.47	80.47
7	510	496.71	−13.29	417.46	−92.54
8	384.59	–	–	416.47	31.88
9	376.89	–	–	–	–
10	465.06	–	–	–	–
11	452.74	–	–	474.66	21.92
12	–	–	–	480	–
Mean (*SD*)	446.24 (47.89)	496.81 (45.44)	32.89 (38.13)	469.67 (41.15)	18.17 (68.92)

SD=standard deviation, D30 = 30 days after transplant, D90 = 90 days after transplant,

diff=difference between baseline and D30 or D60, respectively, − = missing.

Baseline =  6-minute walk test conducted during standard clinical procedures for transplantation, most often at the New Patient Evaluation (−180 to −30 Days prior to transplant). In cases where data from the New Patient Evaluation were missing, baseline is the result obtained from a 6-minute walk test conducted prior to the start of chemotherapy/radiation (−7 to −14 Days before transplant).

### Survival

At 90 days, all but one patient was alive (91.7% survival). Cause of death was multi-organ failure. The 1-year survival rate was 66.7%. Additional causes of death were recurrent disease (*n* = 2) and sepsis (*n* = 1).

## Conclusions

The current study takes an important first step in developing a targeted psychological intervention to optimize HCT outcomes. The premise of the project is that by helping HCT patients and their caregivers adopt an open and receptive posture with respect to unwanted internal experiences (distressing thoughts and feelings) and identify personal values guiding action, patients may be better able to sustain nutrition and physical activity when it is most difficult. This includes, for example, during chemotherapy/radiation induced nausea and fatigue when patients are likely to struggle to eat and walk therefore increasing their risk of excessive weight loss and placement on total parenteral nutrition or critical illness myopathy. Overall, a brief ACT protocol appears to be acceptable and feasible in this patient population, albeit with some manageable complications.

Patients and caregivers were motivated to attend sessions and reported liking the sessions and finding them helpful. The VQ seemed to capture change over time in patients and may have been influenced by transplant outcomes and course, a consideration for future treatment development. A Phase 2 study is needed to determine if decreased values obstruction (or increased psychological flexibility) translates into greater resiliency during the arduous treatment course, and thus better post-transplant outcomes.

In this sample of patients, the majority of patients had improvement in the 6MWT at 90 days. Prior studies have shown a decline in physical function at the 90 day mark following HCT in patients without ACT intervention [38,39]. While the effect of ACT on physical function has not yet been assessed in cancer patients, several studies have shown that ACT may improve physical function as measured by an improvement in two-minute walk test as well as 1-mile walk test time [40,41]. The other outcome measured in our study was weight/BMI. ACT has previously been described as a method to improve nutritional status [42,43], though this is the first study to evaluate its utility in cancer patients undergoing intensive treatment. We found that, although the majority of patients had lost weight at the 90 day mark, most patients only lost between 2 and 5 kg and only 3 patients lost between 9 and 15 kg. This did not differ from studies of patients who underwent HCT but did not receive ACT intervention, as they similarly found that most patients lose between 2 and 5 kg from admission to discharge [35,44]. However, comparison to a control group is required to determine the impact of ACT on nutritional status in patients undergoing HCT.

Caregivers reported low levels of values obstruction at baseline and throughout the duration of the study, relative to their patient counterparts. Their report of obstruction over time was highly variable, with a few caregivers reporting overall improvements and others reporting an increase or no change. This might be because being a caregiver itself is a highly aligned values activity for most individuals. It could also be that other measures would better capture caregiver barriers to engaging in caregiving tasks (e.g., insisting that their loved one to eat or move when they are already uncomfortable) or changes in psychological flexibility in this context. This might include, for example, the Caregiver Inventory, which measures self-efficacy for navigating the demands of caring for the patient with cancer in 4 domains: Managing Medical Information, Caring for Care Recipient, Caring for Oneself, and Managing Difficult Interactions/Emotions [45]. An alternative measure of psychological flexibility might also be better suited to capture changes, such as the CompACT [46]. The CompACT has good psychometric properties and subscales that allow for measurement of separate measurement of three facets of psychological flexibility: openness to experience, behavioral awareness and valued action.

The current study had challenges that are important for future studies. First, it took longer than expected to recruit participants resulting in truncated baseline timelines for some individuals. This was due to the complexities of transplant (e.g., challenges in determining whether transplant is the optimal treatment plan, finding a donor match) and the burden of treatment. Treatment complexity and burden also made data collection challenging and there was significant missing data. Treatment timelines varied due to realities of transplant, unexpected complications and hospitalizations. Verbal feedback from patients and caregivers also suggested that the optimal timing of the intervention may differ by individuals with some patients and caregivers needing more support prior to transplant and others experiencing greater challenges during the post-transplant period.

Participant selection is also a limitation of the study. While all HCT patients were approached about the study, those who decided to participate may have differed in some way from those who did not decide to participate. The results of the current study may not be generalizable to individuals receiving HCT who declined to participate or who may be in other regions of the United States or other parts of the world with varying health care practices.

In conclusion, study findings suggest that ACTivate is acceptable and feasible with preliminary evidence of improvements in psychological flexibility. The protocol was developed with feedback from HCT patients and caregivers and the health care team, and it was trainable to a second interventionist with limited knowledge of the therapeutic approach. This study suggests that larger well-controlled studies may be warranted.

Future studies should include multimodal assessment of psychological and behavioral processes of change and outcomes and might also explore strategies to match dyads to session schedules or otherwise take a more personalized approach given the variability in treatment trajectory. For example, some individuals experience the most pain, discomfort and distress during pre-transplant conditioning, whereas this occurs 2-weeks post-transplant for others, or even during the months following due to unexpected complications (e.g., infection) or reoccurrence of disease.
